# Timing of Influenza A(H5N1) in Poultry and Humans and Seasonal Influenza Activity Worldwide, 2004–2013

**DOI:** 10.3201/eid2102.140877

**Published:** 2015-02

**Authors:** Lizette O. Durand, Patrick Glew, Diane Gross, Matthew Kasper, Susan Trock, Inkyu K. Kim, Joseph S. Bresee, Ruben Donis, Timothy M. Uyeki, Marc-Alain Widdowson, Eduardo Azziz-Baumgartner

**Affiliations:** Centers for Disease Control and Prevention, Atlanta, Georgia, USA (L.O. Durand, P. Glew, D. Gross, S. Trock, I.K. Kim, J.S. Bresee, R. Donis, T.M. Uyeki, M.-A. Widdowson, E. Azziz-Baumgartner);; World Health Organization Regional Office for Europe, Copenhagen, Denmark (D. Gross);; US Naval Medical Research Unit No. 6, Lima, Peru (M. Kasper)

**Keywords:** H5N1, poultry, human, seasonal influenza, global, influenza, viruses, outbreaks, surveillance, biosafety

## Abstract

Co-circulation of influenza A(H5N1) and seasonal influenza viruses among humans and animals could lead to co-infections, reassortment, and emergence of novel viruses with pandemic potential. We assessed the timing of subtype H5N1 outbreaks among poultry, human H5N1 cases, and human seasonal influenza in 8 countries that reported 97% of all human H5N1 cases and 90% of all poultry H5N1 outbreaks. In these countries, most outbreaks among poultry (7,001/11,331, 62%) and half of human cases (313/625, 50%) occurred during January–March. Human H5N1 cases occurred in 167 (45%) of 372 months during which outbreaks among poultry occurred, compared with 59 (10%) of 574 months that had no outbreaks among poultry. Human H5N1 cases also occurred in 59 (22%) of 267 months during seasonal influenza periods. To reduce risk for co-infection, surveillance and control of H5N1 should be enhanced during January–March, when H5N1 outbreaks typically occur and overlap with seasonal influenza virus circulation.

## Abstract 2

Co-circulation of H5N1 in poultry and humans during seasonal influenza epidemic periods signals the need for enhanced surveillance and biosafety measures.

Co-circulation of influenza A viruses in human and animal reservoirs can provide opportunities for these viruses to reassort and acquire genetic material that facilitates sustained human-to-human transmission, a necessary trait of pandemic viruses (*1*). One influenza strain at the forefront of pandemic preparedness planning is highly pathogenic avian influenza (HPAI) A(H5N1) virus. Asian-lineage H5N1 viruses emerged in domestic birds in Southeast Asia in 1996 and are now endemic in 5 countries (*2*). As of December 2013, H5N1 virus outbreaks have been documented among domestic poultry and wild birds in >60 countries (*3**,**4*). The spread of H5N1 among the world’s domestic poultry population increases the risk for H5N1 to infect humans.

Humans are at risk for H5N1 infection if they have direct or close contact with infected domestic poultry, such as by handling sick animals or their byproducts, caregiving, slaughtering, and butchering; infection can also occur through some forms of indirect contact (e.g., proximity to live poultry or wet markets) (*5**,**6*). During 2003–2013, more than 645 human cases of HPAI H5N1 were confirmed; the case-fatality rate was ≈60% (*7*). H5N1 viruses have not yet acquired the ability to be transmitted between humans beyond 3 generations, therefore failing to show sustained human-to-human transmission (*8*). However, these viruses have wide geographic distribution and the potential to reassort with human seasonal influenza viruses. These characteristics mean that clarifying the timing of H5N1 outbreaks among poultry and infections in humans may be useful for prevention and control activities.

Recently published data suggest a seasonal pattern to H5N1 virus infection among domestic and wild birds (*5**,**9**–**13*). Park et al. noted that H5N1 outbreaks among poultry and infections in humans in Southeast Asia occurred in the cooler months during 1997–2006 (*9*). Other studies from Southeast Asia have suggested that decreasing temperatures may correlate with an increase in the number of H5N1 outbreaks among birds (*14**,**15*). Although ecologic studies suggest that H5N1 virus activity may occur during predictable times of the year, a systematic analysis of global poultry and human H5N1 data has not tested this hypothesis. In this study, we explored whether H5N1 outbreaks among domestic poultry and human H5N1 cases occurred in temporal proximity, occurred during certain climate conditions, or overlapped with human seasonal influenza epidemics.

## Methods

### Timing of H5N1 Outbreaks among Poultry

To describe the temporal patterns of H5N1 outbreaks among poultry worldwide, we extracted the number of monthly outbreaks reported by 52 countries as immediate notifications to the World Organisation for Animal Health (OIE) during January 2004–December 2013 (*16*). We used the reported onset month as the occurrence month for each outbreak. Egypt and Indonesia stopped submitting immediate notifications of new H5N1 outbreaks among poultry after H5N1 in poultry was declared endemic: for Egypt in July 2008, and for Indonesia in September 2006. To obtain the number of monthly outbreaks for these countries, we extracted the number from their OIE biannual disease reports. If only the number of new cases but not the number of outbreaks in which they occurred was reported, we classified the month as having 1 outbreak.

For each country and year, we designated the peak month of H5N1 activity as the month that had the largest number of reported H5N1 outbreaks among poultry. Next, we identified the average peak month of H5N1 activity for each country (e.g., if the peak months in a hypothetical country occurred during January 2010, February 2011, and March 2012, then the average peak month during this period would have been February for this country).

### Timing of Human H5N1 Cases and Poultry H5N1 Outbreaks

**Figure 1:**
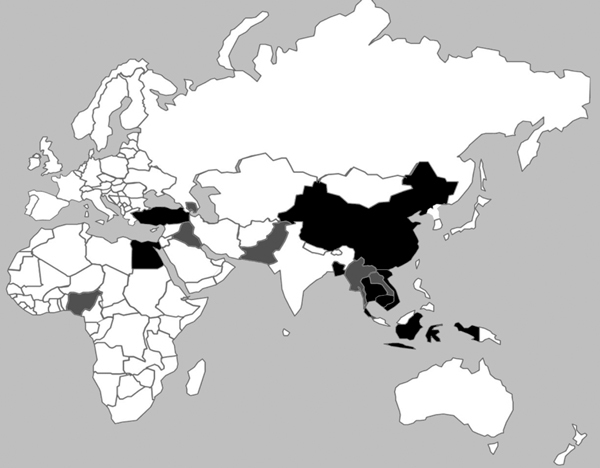
Shading indicates countries that reported confirmed human cases of highly pathogenic avian influenza (HPAI) A(H5N1) infection and outbreaks of H5N1 among poultry during 2004–2013. Black shading indicates the 8 study countries that reported 97% of all human H5N1 cases and 90% of all poultry H5N1 outbreaks: Bangladesh, Cambodia, China, Egypt, Indonesia, Thailand, Turkey, and Vietnam.

We identified the monthly number of human H5N1 cases reported to the World Health Organization (WHO) during January 2004– June 2013. Initially, we obtained data from all 15 countries that reported human H5N1 cases since January 2004 (Figure 1). We then restricted the analysis to the 8 countries that reported 97% of all human H5N1 cases and that reported recurrent H5N1 activity among humans and poultry. To examine the temporal relationship between human H5N1 cases and H5N1 outbreaks among poultry, we compared the number of human H5N1 cases to the number of poultry H5N1 outbreaks for each month from these 8 countries (*7**,**17*). We then analyzed the association of the monthly number of human H5N1 cases and poultry H5N1 outbreaks by using the Spearman coefficient (ρ).

### Timing of Human Seasonal Influenza and H5N1 Cases

To clarify the hypothetical risk for human co-infection with avian and seasonal influenza viruses, we investigated the extent to which human H5N1 cases occurred during months when seasonal influenza viruses were circulating. To determine whether H5N1 and seasonal influenza viruses were identified among humans during the same months, we obtained seasonal influenza surveillance data collected by WHO during 2004–2013 (*18*). We estimated periods of seasonal influenza epidemics by assessing the months during which the proportion of respiratory samples that tested positive for influenza viruses was higher than the annual mean (*19*). We then identified the number of months during which human H5N1 cases and human seasonal influenza epidemics occurred during the same month.

### Ambient Temperature, Human H5N1 Cases, and Poultry H5N1 Outbreaks

To determine whether cool ambient temperature was associated with the number of poultry H5N1 outbreaks, we abstracted monthly mean temperature data for each country from the US National Oceanic and Atmospheric Administration or from the Vietnam General Statistics Office (*20*,*21*). We used linear regression models to determine whether the monthly number of poultry H5N1 outbreaks and number of human H5N1 cases were negatively associated with mean monthly temperature and precipitation (e.g., after accounting for the absolute value of each country’s central latitude [*19*] and 2-way interactions) (*22*).

## Results

### Timing of Poultry H5N1 Outbreaks

During January 2004–June 2013, a total of 12,610 poultry H5N1 outbreaks in 52 countries were reported to the OIE. Fifteen of these countries also reported laboratory-confirmed H5N1 cases in humans to WHO. Of these 15 countries, 8 (Bangladesh, Cambodia, China, Egypt, Indonesia, Thailand, Turkey, and Vietnam) accounted for 11,331 (90%) of worldwide poultry H5N1 outbreaks. These 8 countries served as the basis for our analyses. Four of the 8 countries (Bangladesh, China, Egypt, and Turkey) are classified as Northern Temperate or Subtropical countries because most of their land mass is north of the Tropic of Cancer (latitude 23.27°N). The other 4 countries (Cambodia, Indonesia, Thailand, and Vietnam) are classified as tropical because they are located between the Tropics of Cancer and Capricorn (Table).

**Table:** Summary data for 8 countries that reported 90% of worldwide influenza A(H5N1) outbreaks among poultry and 97% of all human H5N1 cases, January 2004–December 2013*

Country	Geographic region	No. poultry H5N1 outbreaks	No. human H5N1 cases	Peak month for poultry H5N1 outbreaks	Peak month for human H5N1 cases	Peak month for human seasonal influenza cases
China	Northern Temperate	119	44	February	January	February
Egypt	Northern Temperate	2,516	173	March	March	December
Turkey	Northern Temperate	225	12	January	January	March
Bangladesh	Northern Temperate/Tropical	549	7	February	March	July
Cambodia	Tropical	35	47	February	January	October
Indonesia	Tropical	3,555	195	February	January	December
Thailand	Tropical	1,138	25	October	January	September
Vietnam	Tropical	3,194	122	February	January	June

*H5N1 was declared endemic in Indonesia in 2005 and Egypt in 2007. After these dates, these countries did not report outbreaks to the World Organisation for Animal Health on a monthly basis. To obtain the monthly number of outbreaks after countries become endemic, we analyzed their 6-month reports; these reports are made by every country.

**Figure 2:**
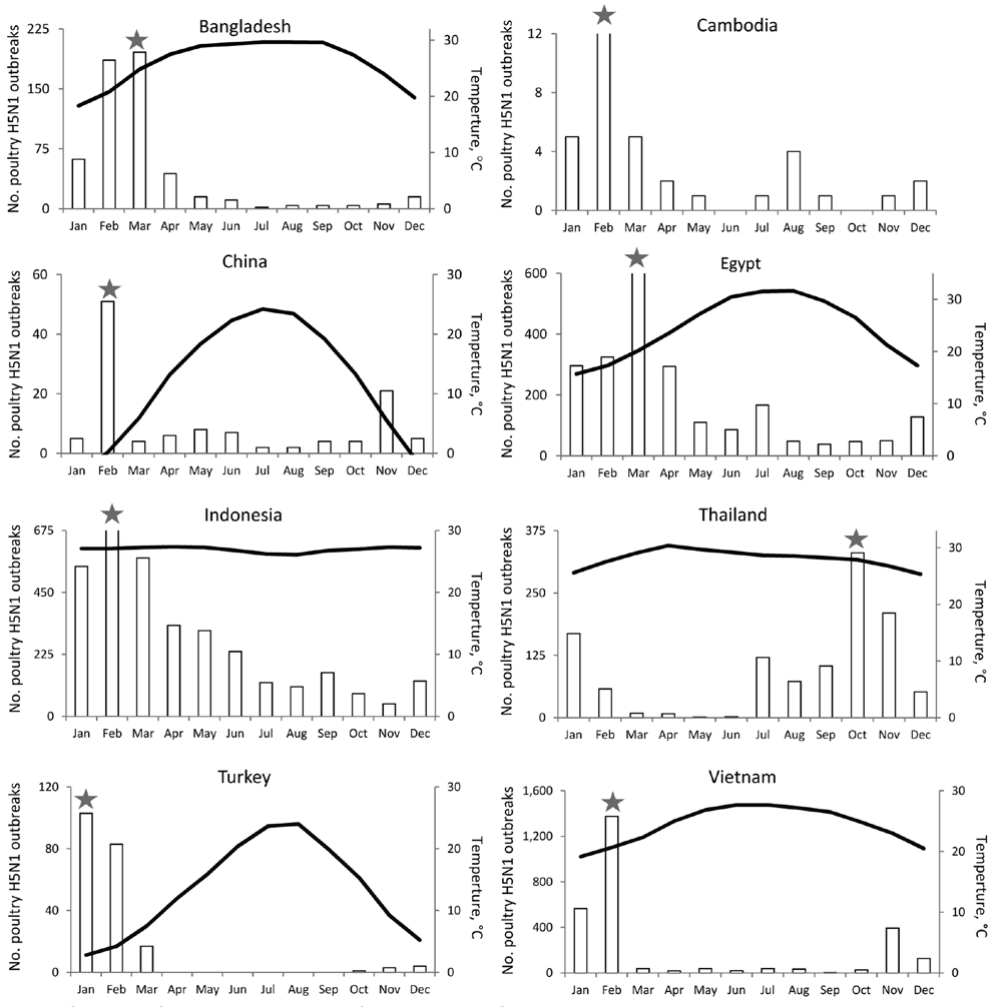
Outbreaks of highly pathogenic avian influenza A(H5N1) infection by month (white bars) and mean temperature (black lines) for the 8 countries that reported 90% of worldwide poultry H5N1 outbreaks during 2004–2013. Stars indicate month with highest average number of outbreaks for each country. Temperature data were not available for Cambodia.

The 8 countries provided a total of 79 whole years (948 months) of data on outbreaks among poultry; 2013 data from Indonesia were not complete and therefore were not included in the analysis. Outbreaks were reported during 39% (365/948) of the study months. Most poultry H5N1 outbreaks (8,616/11,331, 76%) were reported during January–March. February had the highest total number of reported poultry H5N1 outbreaks, 3,118. Figure 2 provides the cumulative number of poultry H5N1 outbreaks reported each month by country and illustrates how these outbreaks clustered during January–March.

### Poultry H5N1 Outbreaks and Human H5N1 Cases

**Figure 3:**
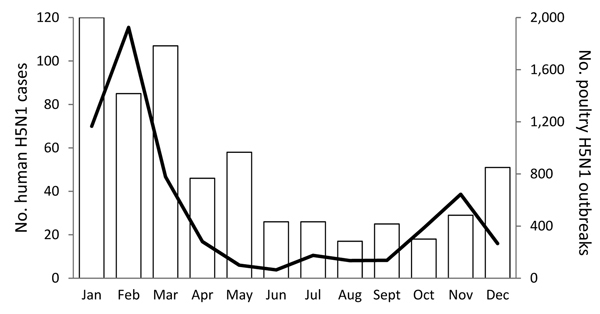
Monthly average number of highly pathogenic avian influenza A(H5N1) infection outbreaks among poultry (black line) and human H5N1 cases (white bars) for 8 study countries (Bangladesh, Cambodia, China, Egypt, Indonesia, Thailand, Turkey, and Vietnam) that reported 90% of all poultry H5N1 outbreaks and 97% of all human H5N1 cases during 2004–2013.

During 2004–2013, the 8 study countries reported nearly all (625/645, 97%) human H5N1 cases (Table); cases were reported in 227 (24%) of the 948 study months. As seen with H5N1 outbreaks among poultry, half of human cases (313/625, 50%) occurred during January–March, and January had the highest number of reported H5N1 cases among humans (ρ = 0.8, p = 0.004) (Figures 2, 3). At least 1 human case of H5N1 infection occurred during 168 (45%) of the 374 months in which ≥1 outbreak in poultry occurred, compared with 59 (10%) of 574 months without outbreaks among poultry (relative risk = 5, p<0.001). After their initial identification, poultry H5N1 outbreaks and human H5N1 cases recurred concurrently during October–March in 24 (34%) of 70 study year equivalents.

### Human H5N1 and Seasonal Influenza Activity

**Figure 4:**
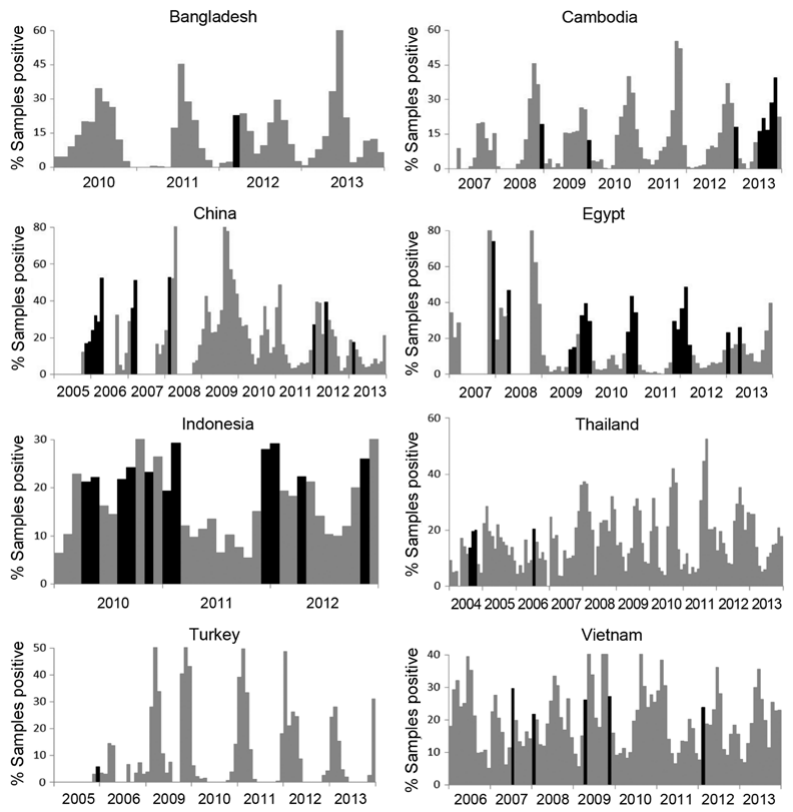
Human cases of highly pathogenic avian influenza A(H5N1) infection and seasonal influenza rates for 8 study countries, by month. Bars indicate the proportion of human respiratory samples that tested positive for seasonal influenza viruses; black bars indicate months during which seasonal influenza was epidemic and a human H5N1 case was reported. Years covered for each country are provided along *x*-axes.

Seasonal influenza was epidemic in 267 (30%) of 888 months for which data were available. Human H5N1 cases occurred in 59 (22%) of these 267 months (Figure 4).

### H5N1 Outbreaks in Poultry during Colder Temperatures

In each of the 8 countries, the number of poultry H5N1 outbreaks was highest during months with the lowest average ambient air temperature (Figure 2). In our regression model, colder ambient temperature was also ecologically associated with an increase in the number of poultry H5N1 outbreaks and human cases. For each decrease in temperature by 3.3°C, 1 additional poultry H5N1 outbreak was reported (p = 0.004), and for each decrease in temperature by 0.3°C, 1 additional H5N1 human case was reported (p<0.001). Precipitation was co-linear to temperature, however, and was dropped from the analysis.

## Discussion

Our study reaffirms that, in Southeast Asia, H5N1 outbreaks among poultry and human H5N1 cases often occur seasonally, during months when temperatures are relatively cool. Even when accounting for H5N1-endemic countries outside Southeast Asia, most (>50%) poultry H5N1 outbreaks and human H5N1 cases of H5N1 infection occurred during January–March. Human and animal health officials in affected countries should consider exploring the value of enhanced surveillance, flock biosecurity, and live bird market disinfection and/or rest days during periods when H5N1 viruses are typically detected (*22**–**24*). 

Our analysis of 2004–2013 data from 8 countries also suggests that lower ambient temperatures are associated with H5N1 outbreaks among poultry, even though half of our data came from tropical countries, where annual temperature variations are often small. These results are similar to those described by Park and Glass, who observed poultry H5N1 outbreaks during 1997–2006 in Southeast Asia and China and concluded that these outbreaks most often occurred during colder months (*10*). Other studies have found similar associations (*5**,**25**,**26*). A decrease in temperature can make poultry more susceptible to H5N1 because lower ambient temperature can decrease poultry immunity (*27**–**29*). Moreover, cold weather may enable prolonged viral survival in the secretions and feces of infected poultry, and anticipation of seasonal holidays (e.g., Chinese New Year) often results in increases in population density of domestic poultry and in trafficking of poultry (*27**–**34*).

Human H5N1 cases were almost 5 times more common in months during which poultry H5N1 outbreaks occurred. These findings reaffirm reports that human H5N1 virus infection is typically preceded by exposure to sick or dead poultry (*35*) and suggest that human and animal health officials in affected countries should explore the effectiveness of education and outreach efforts before and postexposure prophylaxis during anticipated H5N1 epidemic periods. These efforts could further enhance surveillance of H5N1 in poultry and lead to more prompt culling of poultry when H5N1 viruses are detected, thus helping to increase flock biosecurity and enable timely education and outreach efforts. For example, affected countries may want to evaluate the cost-effectiveness of targeted risk communication campaigns delivered before peak months of H5N1 infection to promote the avoidance of sick or dead poultry, the importance of hand washing, and the appropriate use of personal protective equipment among subpopulations that frequently are in contact with poultry. These efforts could help mitigate the transmission of H5N1 viruses among poultry and the spread of the virus to human populations (*22**–**24*).

Our data also suggest that one fifth of human H5N1 cases occurred in months during which seasonal influenza was epidemic. Concurrent H5N1 and human seasonal influenza activity provides opportunities for humans and other animals (e.g., swine) to become co-infected with these co-circulating viruses and for the viruses to reassort. Reassortment may generate novel influenza A virus strains with the ability to cause sustained human-to-human transmission.

Our findings support and demonstrate the feasibility of integrating human and animal surveillance for avian influenza and other zoonotic diseases. Combining the expertise from multiple surveillance systems can increase the detection rate of H5N1 cases in humans from 57% to 93% and of epizootics from 40% to 53% (*36*). This recommendation for an integrated H5N1 surveillance approach enables better prediction of disease risk and identification of outbreaks (*37*). Such approaches can assist in filling in gaps in human and animal surveillance systems (*36**, **37*).

This study has several limitations. H5N1-endemic countries are not required to report immediate notifications. H5N1 reports also often are limited because of a lack of resources for surveillance, laboratory capacity to confirm H5N1, and, often, the political will to report outbreaks in poultry that may result in substantial economic losses (*38*). No formal case definition for a poultry H5N1 outbreak has been developed. During our study period, surveillance practices for human and poultry H5N1 and human seasonal influenza were not standardized; in fact, outbreaks among poultry might be more likely to be reported during periods when human H5N1 cases occurred. Further standardization of surveillance and reporting of influenza outbreaks in humans and animals might help clarify the epidemiology of these viruses (*19*).

Health authorities in H5N1 virus–affected countries should consider enhanced surveillance for H5N1 viruses among domestic poultry and humans in anticipation of cooler months, when H5N1 virus outbreaks are most likely to occur. Although year-round surveillance is important for the identification of novel strains of influenza, laboratorians should emphasize the identification, reporting, and sharing of nonsubtypable influenza A viruses during January–March. Public and animal health authorities may consider enhancing animal market surveillance during this time frame and encourage the use of personal protective equipment, disinfectant, and other resources for containment and mitigation in anticipation of increased H5N1 activity among poultry (*22**–**24*). These lessons may also be relevant to the management of avian influenza A(H7N9) virus outbreaks in China, which have occurred during similar times of the year.
